# Tracking Components of Bilingual Language Control in Speech Production: An fMRI Study Using Functional Localizers

**DOI:** 10.1162/nol_a_00128

**Published:** 2024-06-03

**Authors:** Agata Wolna, Jakub Szewczyk, Michele Diaz, Aleksandra Domagalik, Marcin Szwed, Zofia Wodniecka

**Affiliations:** Institute of Psychology, Jagiellonian University, Kraków, Poland; Donders Institute for Brain Cognition and Behaviour, Radboud University, Nijmegen, The Netherlands; Max Planck Institute for Psycholinguistics, Nijmegen, The Netherlands; Social, Life, and Engineering Sciences Imaging Center, Pennsylvania State University, Pennsylvania, USA; Centre for Brain Research, Jagiellonian University, Kraków, Poland

**Keywords:** bilingual language control, bilingualism, cognitive control, precision fMRI, speech production

## Abstract

When bilingual speakers switch back to speaking in their native language (L1) after having used their second language (L2), they often experience difficulty in retrieving words in their L1. This phenomenon is referred to as the *L2 after-effect*. We used the L2 after-effect as a lens to explore the neural bases of bilingual language control mechanisms. Our goal was twofold: first, to explore whether bilingual language control draws on domain-general or language-specific mechanisms; second, to investigate the precise mechanism(s) that drive the L2 after-effect. We used a precision fMRI approach based on functional localizers to measure the extent to which the brain activity that reflects the L2 after-effect overlaps with the language network (Fedorenko et al., 2010) and the domain-general multiple demand network (Duncan, 2010), as well as three task-specific networks that tap into interference resolution, lexical retrieval, and articulation. Forty-two Polish–English bilinguals participated in the study. Our results show that the L2 after-effect reflects increased engagement of domain-general but not language-specific resources. Furthermore, contrary to previously proposed interpretations, we did not find evidence that the effect reflects increased difficulty related to lexical access, articulation, and the resolution of lexical interference. We propose that difficulty of speech production in the picture naming paradigm—manifested as the L2 after-effect—reflects interference at a nonlinguistic level of task schemas or a general increase of cognitive control engagement during speech production in L1 after L2.

## INTRODUCTION

In their daily lives, bilinguals must face a constant challenge related to the nonselectivity or constant co-activation of their two languages (Costa et al., 1999; Kroll et al., 2006). To cope with this problem, bilinguals need an efficient system of control that resolves interference between the competing languages (Green, 1998), or a selection mechanism (Blanco-Elorrieta & Caramazza, 2021) that allows them to efficiently use the intended language. In this study we aimed to characterize the neurocognitive mechanisms of control that are engaged in bilingual speech production by capitalizing on the advantages of the *functional localizer* approach. To this aim, we explored how the control mechanisms affect bilingual speech production by looking at the *L2 after-effect*: a difficulty in native language (L1) production observed after prior use of a second language (L2).

### Bilingual Language Control: Domain General or Domain Specific?

One of the most fundamental questions regarding the complex system that bilinguals use to control their two languages is whether it is based on a set of mechanisms specifically tailored to handle language (so called domain-specific, or language-specific mechanisms), or whether bilingual language control is achieved by domain-general cognitive control mechanisms (Green & Abutalebi, 2013; Wu et al., 2019; for a review see Calabria et al., 2018).

So far, most evidence for the engagement of domain-general control mechanisms in bilingual language production comes from behavioral studies in which bilinguals were required to switch between their languages. The overall conclusion from these experiments is that switching between two languages incurs additional processing costs (Costa & Santesteban, 2004; Gollan & Ferreira, 2009; Meuter & Allport, 1999; Verhoef et al., 2009; for review see Bobb & Wodniecka, 2013; Declerck et al., 2020; Declerck & Koch, 2023; Declerck & Philipp, 2015). This additional cognitive effort has also been observed as increased neural activation in structures associated with broadly defined cognitive control mechanisms (e.g., Abutalebi & Green, 2007; Coderre et al., 2016; De Baene et al., 2015; Wu et al., 2019; for reviews see Abutalebi & Green, 2016; Luk et al., 2012; Sulpizio et al., 2020). It has been argued that bilingual language control is supported by domain-general mechanisms because task switching (which recruits domain-general control) and language switching share a number of computational processes, for example, context monitoring, reconfiguring task sets, engaging and disengaging from a task, suppressing the interference between competing representations and selective response inhibition (Green, 1998; Green & Abutalebi, 2013; Wu et al., 2019). This is also supported by research showing that the brain networks that support language switching and task switching overlap (Blanco-Elorrieta & Pylkkänen, 2016; Coderre et al., 2016; De Baene et al., 2015; Luk et al., 2012; Mendoza et al., 2021; Nee et al., 2007; Niendam et al., 2012; Weissberger et al., 2015; Wu et al., 2019).

On the other hand, the overlap between brain networks activated by language- and task-switching may not provide conclusive evidence for the domain-general nature of bilingual language control. First, language switching also engages brain structures that are not implicated in nonlinguistic switching tasks (De Baene et al., 2015; Mendoza et al., 2021; Weissberger et al., 2015). Second, the overlap between the brain networks recruited by language- and task-switching may result from idiosyncratic properties of the switching task itself rather than bilingual language control. More specifically, it can be argued that language switching is an artificial task in the sense that it requires changing languages very frequently and depending on external cues, while switching in natural contexts is largely dictated by the speaker (i.e., internal cues; for discussion on how cues affect switch costs, see Blanco-Elorrieta & Pylkkänen, 2017, for natural vs. artificial cues and Gollan et al., 2014, and Gollan & Ferreira, 2009, for voluntary vs. forced cues). As such, a language switching task may require using a very specific task set and keeping both languages co-activated. Such extra demands may recruit mechanisms engaged in performing tasks that are untrained, difficult, or not automated (Chein & Schneider, 2005; Crittenden & Duncan, 2014; Shashidhara et al., 2019), and these types of tasks are known to reliably activate the domain-general multiple demand (MD) network. What is more, bilinguals rarely switch between their languages on a word-by-word basis (Wodniecka, Casado, et al., 2020; see also Bobb & Wodniecka, 2013), and forced switching is related to greater engagement of cognitive control than voluntary switching (for behavioral evidence see Gollan et al., 2014; Gollan & Ferreira, 2009; for fMRI evidence see Reverberi et al., 2018; Zhang et al., 2015).

### Digging Deeper—Specific Processes Involved in Bilingual Language Control

Besides the more general question of whether bilinguals rely on domain-general or language-specific networks when using their two languages, another unresolved issue is what specific processes are involved in bilingual language control. Models of bilingual language production assume that each language of a bilingual speaker is subserved by a set of different lexical, phonological, and articulatory representations (e.g., Blanco-Elorrieta & Caramazza, 2021; Costa, 2005; La Heij, 2005). Successful production requires using representations that belong to the target language but not to the non-target language. In the case of bilingual speakers (especially L1-dominant speakers), it is typically assumed that using their L1 does not require additional control over representations of their L2 because L1 representations are readily available and receive no interference from L2 representations. However, when an L1-dominant bilingual wants to use their weaker L2, the representations of L2 are less active and receive interference from strongly activated L1 representations, so accessing them may require additional control processes. The most prominent theory of bilingual language control proposes that resolution of between-language interference is implemented by active inhibition of L1 representations (Green, 1998); however, it does not specify at which level of word processing (e.g., lexical, phonological, articulatory) inhibition is implemented. On the other hand, other theoretical models argue that inhibition is not necessary for the successful selection of the target language (Blanco-Elorrieta & Caramazza, 2021; Costa, 2005; Koch et al., 2010). For example, the persisting activation account assumes that when a bilingual wants to use the weaker L2, its representations receive additional activation, which allows them to surpass the level of activation of the stronger L1 (for a review see Koch et al., 2010). A recent extension of the activation-based account proposed that activation of target representations does not operate on a whole-language level (i.e., it does not apply to all items of L1 or L2) but is rather a sum of activations driven by different factors, such as baseline word frequency, recency of use, and communicative context (Blanco-Elorrieta & Caramazza, 2021). As such, both bilingual and monolingual speech production can be explained by the same selection mechanisms that select the most active representation at a given moment.

### The L2 After-Effect as a Window to Study Neurocognitive Mechanisms of Bilingual Language Control

As has been already indicated, the lion’s share of evidence on the involvement of control in bilingual language production comes from the language-switching paradigm (for a recent review see Sulpizio et al., 2020), which—as we argued above—has several important limitations. Some of these limitations are circumvented in a blocked language picture-naming task that does not require as frequent switches between languages as the language switching task and yet allows control mechanisms involved in bilinguals’ language production to be tested. Several studies have observed that completing a task in L2 results in a subsequent slow-down of naming or an increase of errors committed in L1 (Branzi et al., 2014; Degani et al., 2020; Gollan & Ferreira, 2009; Van Assche et al., 2013; Wodniecka, Szewczyk, et al., 2020). This difficulty in using L1 after a short exposure to L2 has been referred to as the L2 after-effect. Any task involving language production in L2 can lead to the L2 after-effect: simply reading a list of words aloud (Degani et al., 2020), naming a set of pictures (Branzi et al., 2014, 2016; Gollan & Ferreira, 2009; Guo et al., 2011; Wodniecka, Szewczyk, et al., 2020) or coming up with words belonging to a given semantic category (Van Assche et al., 2013).

Available theories of bilingual speech production provide different explanations of the L2 after-effect. According to inhibition-based accounts, the retrieval difficulty that speakers experience in L1 after using L2 is a lingering consequence of the strong inhibition that had to be applied to L1 representations during L2 use (Green, 1998; Guo et al., 2011; Misra et al., 2012). Under the inhibitory accounts, resolution of the interference that drives the L2 after-effect relies on a set of domain-general mechanisms (e.g., Abutalebi & Green, 2016; Green, 1998; Green & Abutalebi, 2013). An alternative proposal assumes that the L2 after-effect is driven by increased interference between the representations of L1 and L2 due to the carry-over effects of increased L2 activation (Blanco-Elorrieta & Caramazza, 2021; Branzi et al., 2014, 2016; Koch et al., 2010).

To date, the origin of the L2 after-effect has been explored in three neuroimaging studies using functional magnetic resonance imagine (fMRI). They revealed that the difficulty in word retrieval in L1 after using L2 results in increased engagement of brain regions often linked to domain-general control mechanisms (Branzi et al., 2016; Guo et al., 2011; Rossi et al., 2018) such as interference suppression (left middle frontal gyrus, left inferior parietal gyrus; Guo et al., 2011), visual attention shifts (left parietal cortex; Guo et al., 2011), articulation (right postcentral gyrus; Guo et al., 2011), response inhibition (right inferior frontal cortex [Branzi et al., 2016]; right frontal pole and right superior parietal lobule [Rossi et al., 2018]), response selection (left prefrontal cortex; Branzi et al., 2016), attention (bilateral inferior parietal lobules; Branzi et al., 2016), and conflict monitoring and resolution (bilateral anterior cingulate cortex; Rossi et al., 2018). While most of these structures have been linked to several different domain-general control mechanisms, some structures are thought to support language-specific processing as well as articulation. As such, the results of previous studies do not unanimously identify the mechanisms that drive the L2 after-effect.

### Capturing the Neural Basis of the L2 After-Effect: Methodological Challenges

Importantly, three studies that explored the L2 after-effect were not free of methodological challenges. Two out of three studies report neural contrasts that involved different samples of participants (Guo et al., 2011; Rossi et al., 2018). This could be problematic as the engagement of language control mechanisms may be dependent on some aspects of language experience (see Casado et al., 2022, for evidence of the influence of language balance on the magnitude of engagement of global language control). What is more, a study by Rossi et al. (2018) compared a sample of bilinguals to monolinguals, but this does not make it possible to disentangle the effects of previous use of L2 from more general differences between bilingual and monolingual language processing. Branzi and colleagues (2016) addressed these limitations by comparing preceding context effects within the same bilingual participants; however, in this study, the baseline L1 block, which was compared to L1 after L2 in order to estimate the L2 after-effect, was in some participants preceded by a task in L2. Since the L2 after-effect is relatively long-lasting (at least 5 min; see Branzi et al., 2014; Casado et al., 2022; Wodniecka, Szewczyk, et al., 2020; for review see Wodniecka, Casado, et al., 2020), a baseline for comparison (i.e., L1 naming) should not be preceded by a task in L2. It is therefore possible that the observed L2 after-effect was confounded by factors such as training, fatigue, or accumulating semantic interference (Runnqvist et al., 2012; for a discussion of how trial-related factors can affect the measurement of L2 after-effect see Casado et al., 2022).

Moreover, the previous studies relied on group-level fMRI analysis. This approach focuses on the commonalities in activations between participants but is less sensitive to individual variability in brain organization, which is especially important among bilinguals whose variable language experience (related to a number of factors, such as immersion, age of acquisition, proficiency, daily use of language), may translate into changes in the functional and structural organization of the brain (e.g., DeLuca et al., 2019, 2020; Fedeli et al., 2021). What is more, while previous studies undoubtedly identified brain structures engaged in the L2 after-effect, the interpretations regarding the precise mechanisms represented by the observed brain response were not directly tested and were proposed based on the function assigned to a given anatomical structure (the so-called *reverse inference* problem; Poldrack, 2006; for a discussion of issues related to this approach in studying language see Fedorenko, 2021). It has been shown that macroanatomical cortical areas are characterized by vast interindividual variability, which means that the link between anatomical structure and functional organization is not straightforward, especially for high-level cognitive processes such as language (Frost & Goebel, 2012; Tahmasebi et al., 2012; for a discussion see Fedorenko, 2021; Michon et al., 2022).

### Current Study

In the current study, we explored the nature of bilingual language control by looking at the brain basis of the L2 after-effect. To address the limitations of experimental designs previously used to measure the L2 after-effect, we used a within-participant design that carefully controlled for the effects of the preceding language context as well as for possible effects of task order and fatigue. We had two specific goals: first, to determine to what extent the control mechanisms engaged in bilingual language control are domain-general versus language-specific; second, to identify the specific process that may underlie difficulties in L1 production following L2 use, namely lexical retrieval difficulty (Casado et al., 2022), articulatory difficulty (Guo et al., 2011), and increased interference between languages (Branzi et al., 2014). We addressed these questions by using fMRI and a set of functional localizers, which—to the best of our knowledge—is an approach that has not been used before in studies exploring the mechanisms of language control in bilinguals. A crucial advantage of functional localizers is that they allow the modeling of brain responses at a subject-specific level; this increases the sensitivity of the statistical analyses and models interindividual variability better than typical group-level analyses.

To explore the domain-specificity or domain-generality of bilingual language control mechanisms engaged in the L2 after-effect, we used two well-established functional localizers that make it possible to track down subject-specific networks that correspond to the language system (Fedorenko et al., 2010; Malik-Moraleda et al., 2022) and the multiple demand network (Duncan, 2010; Fedorenko et al., 2013). The language localizer is a well-established paradigm that allows the identification of a robust network in the brain spanning the frontal, temporal, and parietal regions and supporting the processing of language (e.g., Fedorenko, 2021; Fedorenko et al., 2010; Fedorenko et al., 2011; Malik-Moraleda et al., 2022). This network has a robust specificity for language; it is engaged during reading (e.g., Fedorenko et al., 2010), listening (e.g., Pallier et al., 2003; Scott et al., 2017), and production (Hu et al., 2023; Liljeström et al., 2015), but it shows little engagement in nonlinguistic tasks (Benn et al., 2023; Chen et al., 2023; Fedorenko et al., 2011; Ivanova et al., 2021) or social reasoning (Shain et al., 2023). Importantly, even though the language network is usually localized using a comprehension-based task it is equally sensitive to language production (Hu et al., 2023).

The multiple-demand localizer, on the other hand, allows the identification of another robust network in the brain that responds to increased task demands across many different types of tasks (Assem, Blank, et al., 2020; Braver et al., 2003; Cole & Schneider, 2007; Dosenbach et al., 2006; Duncan, 2010; Fedorenko et al., 2011; Fedorenko et al., 2013; Miller & Cohen, 2001). Activity within this network has been shown to decrease with the increasing automatization of task performance (Chein & Schneider, 2005) and increase as a function of task complexity (Crittenden & Duncan, 2014; Fedorenko et al., 2013; Shashidhara et al., 2019), time pressure, and reward (Shashidhara et al., 2019). Importantly, the frontoparietal MD network shows little or no domain-specificity (Assem, Glasser, et al., 2020); it is robustly activated in a range of different linguistic and nonlinguistic cognitive tasks (Dosenbach et al., 2006; Fedorenko et al., 2013; Shashidhara et al., 2019), including low-level perceptual tasks (Cole & Schneider, 2007).

While both the language and the MD networks show vast cross-subject variability (Fedorenko et al., 2010; Smith et al., 2021), they show a clear functional and topographic dissociation on a single-subject level (e.g., Blank et al., 2014; Diachek et al., 2020). As such, if brain activity corresponding to the L2 after-effect is found within one of these two networks, it will provide straightforward evidence for the engagement of domain-general or language-specific mechanisms.

To investigate the specific mechanisms that drive the L2 after-effect, we used three additional localizers that focused on lexical access (a localizer based on the verbal fluency task), articulation (a localizer based on a simple articularion task), and lexical interference (a localizer based on the Stroop task). Each of them tapped into one of the previously described mechanisms that possibly underlies the L2 after-effect. The Stroop task (Stroop, 1935) was used to capture brain structures engaged in the resolution of interference and inhibition of non-target responses in speech production (Diamond, 2013). An overlap between these structures and those involved in the L2 after-effect would provide an argument that the L2 after-effect is driven by increased interference between the two languages.

The verbal fluency task was used to identify brain regions that are sensitive to the difficulty of lexical access. As previously discussed, difficulties in lexical access seem to be one of the most parsimonious explanations of the L2 after-effect (Casado et al., 2022; Wodniecka, Szewczyk, et al., 2020). The verbal fluency task is thought to engage cognitive mechanisms that support word retrieval (Birn et al., 2010; Friesen et al., 2021; Shao et al., 2014). Specifically, the verbal fluency task was shown to tap into the efficiency of the lexical search and selection processes (Shao et al., 2014) as well as executive function mechanisms engaged in word retrieval (Friesen et al., 2021; Martin et al., 1994). Therefore, if L1 retrieval difficulty after L2 is due to L1 lexical access being hampered, then the brain activity corresponding to the L2 after-effect should overlap with the network identified using a verbal fluency localizer. Finally, verbal fluency performance has been shown to depend either on individual differences between subjects that are related to vocabulary size and working memory capacity, or on the mere speed of information processing (Unsworth et al., 2011). Therefore, a subject-specific localizer based on a verbal fluency task allows us to better capture the interindividual variability in lexical retrieval between subjects.

Finally, to test the extent to which the L2 after-effect is driven by the articulatory difficulty of word production in L1, we used a localizer that allows us to identify brain regions specifically linked to articulation (Basilakos et al., 2018). This localizer was based on a contrast between articulating syllables and performing a simple motor sequence (finger tapping). An fMRI experiment that examined the neural basis of the L2 after-effect suggested that speaking in L1 after being exposed to L2 entails the engagement of additional articulatory resources (Guo et al., 2011). Moreover, more recent behavioral evidence (Broos et al., 2018) suggests that greater difficulty in speaking L2 compared to L1 only affects the late articulatory stage; if so, it is also plausible that exposure to L2 results in articulatory difficulties for subsequent L1 use.

## MATERIALS AND METHODS

### Participants

Forty-two Polish–English late bilinguals took part in the study. One participant was excluded due to excessive head motion (>2 mm) in the main fMRI task, resulting in a final sample of 41 participants (31 females, 10 males, mean age = 23.29, *SD* = 3.24, range: 19–32). All participants were Polish native speakers who learned English as their second language. To participate in the experiment, they had to declare sufficient proficiency in English (B2 or higher) and obtain at least 20/25 points on the General English Test by Cambridge Assessment (mean score = 21.32, *SD* = 1.94). In the behavioral session, the participants’ proficiency in English was assessed again using LexTale (mean score = 71.58%, *SD* = 9.91%; Lemhöfer & Broersma, 2012). In addition, participants completed a questionnaire in which they rated their proficiency in all known languages with regards to reading, writing, listening, speaking, and accent; participants also gave information on their daily use of all languages and the age at which they started to learn each of them. Detailed information on the participants’ proficiency, age of acquisition, and daily use of languages can be found in Table 1. All participants gave written consent for participation in the study. The study was approved by the Ethics Committee of the Institute of Psychology of the Jagiellonian University concerning experimental studies with human subjects.

**Table 1.** Language experience of participants

Language	L1 (Polish)	L2 (English)	L3 (various)	L4 (various)
*n*	41	41	36	17
**Self-rated proficiency (1–10)**				
Reading	9.72	4.34	2.76	1.79
Listening	9.62	4.10	2.45	1.84
Writing	9.26	3.81	2.21	1.48
Speaking	9.49	3.89	2.48	1.68
Accent	9.56	3.45	2.45	1.65
**Language learning**				
Age of acquisition onset of learning	0.00	6.44	14.34	15.24
Age of acquisition onset of using	3.17	9.15	15.41	16.88
Years of formal education	14.72	11.56	4.03	4.18
**Daily use of languages**				
Passive	63.26%	31.79%	4.03%	3.25%
Active	81.31%	16.31%	2.06%	1.31%
**Verbal fluency (mean number of words produced)**				
Category	20.04	13.36	–	–
Letter	12.64	10.62	–	–

*Note*. Information on self-rated proficiency, language learning and daily use of languages is given for all languages that participants declared to know. L1 always refers to Polish and L2 to English. L3 and L4 were a variety of different languages (incl. German, French, Italian, Spanish, Russian, Czech, Japanese, Korean, Norwegian, Latin and Esperanto). Not all the participants reported knowing an L3 or L4.

### Study Design

**Figure 1.**
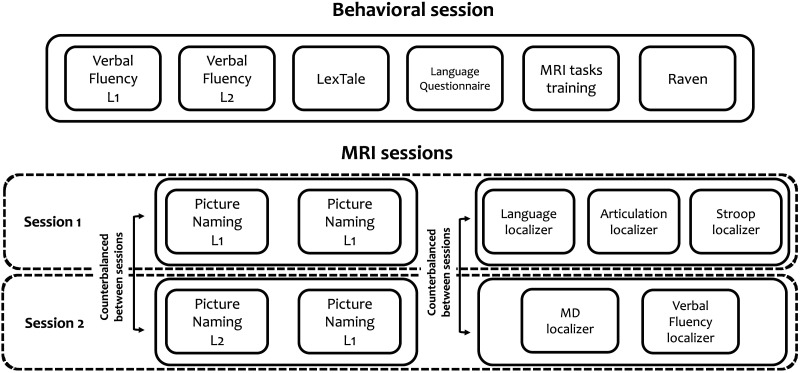
Summary of the design of the behavioral and MRI sessions. In the behavioral sessions, all participants completed the tasks in a fixed order. In the MRI sessions, the order of conditions completed in the first and second sessions (experimental manipulation with L1 or L2) was independently counterbalanced between participants; the set of localizer tasks assigned to each session was also counterbalanced between participants. L1 = native language, L2 = second language, MRI = magnetic resonance imaging, MD = multiple demand.

The experiment consisted of three testing sessions: one behavioral and two MRI scanning sessions. The design of the behavioral and MRI sessions is presented in detail in Figure 1, and the details of each task are discussed in the Design section.

In the behavioral session, participants completed a series of tasks, including semantic and phonetic verbal fluency in L1 and L2 (see details below), LexTale in English (Lemhöfer & Broersma, 2012), a language questionnaire, and a short version of Raven’s Progressive Matrices test (only uneven items) as a measure of a nonverbal intelligence. The behavioral session also included a short training session on the tasks to be performed in the MRI sessions.

During the MRI sessions, participants completed the main task, namely picture naming, and a series of localizer tasks. The main task was split between two sessions, with one condition (L1 or L2 in the first block) completed in the first session and the other condition completed in the second session, with the order counterbalanced across sessions. Localizer tasks included an auditory language localizer task (Malik-Moraleda et al., 2022; Scott et al., 2017), an articulation localizer, a Stroop task, a visual working memory task corresponding to the MD localizer (Fedorenko et al., 2013), and a verbal fluency task (order counterbalanced; see Figure 1 for details). In tasks requiring overt spoken responses (i.e., picture naming, Stroop task, articulation task, and verbal fluency) participants’ responses were recorded with FOMRI III, which is an MR-compatible, fiber-optic microphone system (Optoacoustics, Or-Yehuda, Israel). Participants were instructed to minimize jaw movements while giving the spoken responses, which they had practiced during the behavioral session.

### Materials and Procedure

#### Behavioral session

##### Verbal fluency task.

In this task, participants were asked to produce as many words belonging to a given category (fruits or animals) or beginning with a given letter (B or M) as they could within a 1 min time limit. Cues were counterbalanced between languages. Each participant first completed the task in Polish (with one category and one letter) and then in English (also with one category and one letter). Each cue (letter or category) was displayed on the screen for 1 min, during which participants provided verbal responses that were recorded with the microphone. This task was used as a complementary measure of relative proficiency in L1 and L2; the mean number of words produced for letter and category tasks in each language is reported in Table 1.

##### LexTale.

In the LexTale task, participants were instructed to say whether the string of letters presented on a screen corresponded to an existing word in English. The response (yes/no) was provided via a keyboard. Each word or nonword appeared in the center of the screen and remained there until the participant responded. The task used the same words and nonwords as the original LexTale task (Lemhöfer & Broersma, 2012).

##### Language questionnaire.

In the language questionnaire, participants were asked to respond to four categories of questions. The first category concerned proficiency in the native and foreign languages that the participants knew. It included a self-rating scale on the five language skills (reading, writing, listening, speaking, and accent) and questions concerning the age of acquisition. The second category included questions on patterns of language use: the participants were asked to estimate how much time during a typical day they actively (i.e., speaking, writing) and passively (i.e., watching movies, reading, listening to music/radio/podcasts) use the languages they know. In the third category, participants were asked to provide information about language use contexts: home, work, school, and free time. Specifically, they were asked to estimate the number of hours that they use each language they know in each of these contexts. The fourth category of questions referred to demographic information, such as age, gender, and education. Filling out the entire questionnaire took approximately 15–20 min.

##### MRI task training.

In this part of the behavioral session, participants completed short versions of the tasks that they were going to perform during the MRI session. (For details of the tasks see the MRI session design.) Each task lasted approximately 1–2 min and was repeated if a participant asked for more practice. In tasks requiring overt responses, participants were asked to practice minimizing jaw movements while speaking.

##### Raven test.

In this task, participants completed a shortened version of Raven’s Advanced Progressive Matrices test (only odd-numbered items). They had 20 min to complete as many matrices as they could. The test was used in a paper–pencil format and was aimed to provide a measure of fluid intelligence.

#### MRI sessions—the main task

In the main task, participants were asked to overtly name pictures of objects in four task blocks: two blocks in each session. One task block corresponded to one functional run. The first block of each session corresponded to one of the experimental manipulations: exposure to L1 (which required naming pictures in L1) or exposure to L2 (which required naming pictures in L2). The order of experimental manipulation blocks was counterbalanced between participants; that is, some of them started with the L2 exposure conditions in the first session and completed the L1 exposure condition in the second session, and some of them started with the L1 exposure condition in the first session and completed the L2 exposure condition in the second session. In the second block of each session, participants were asked to name pictures in L1. Each block contained 55 colored pictures representing objects (from the cross-linguistic lexical tasks, or CLT, database; Haman et al., 2015; Wolna et al., 2023). Pictures were not repeated between blocks. Each block started with a fixation cross (“+”) displayed on a screen for 7,000 ms. Subsequently, 55 pictures were presented following a rapid event-related design. Each picture was displayed on a white background for 2,000 ms and was followed by a fixation cross, jittered at an interstimulus interval ranging from 607 ms to 10,144 ms (M = 3,091 ms, *SD* = 2,225 ms). The order of picture presentation and interstimulus intervals was optimized using optseq2 (Dale, 1999). Finally, at the end of each block, a fixation cross was presented for 8,000 ms. Overall, each block of the picture naming task lasted 295 s.

#### MRI sessions—localizer tasks

After completing the main task, in each session participants completed two or three localizer tasks. Each localizer consisted of two functional runs.

##### Language localizer.

Participants listened to fragments of *Alice in Wonderland* in L1 and distorted speech recordings in which it was impossible to understand what the speaker was saying. The task followed the design introduced by Scott et al. (2017) and Malik-Moraleda et al. (2022), in which the localizer contrast was based on the intact > distorted speech condition contrast. The intact condition consisted of short passages from *Alice in Wonderland* read by the native speaker. The distorted speech condition was created based on the intact condition but sounded like speech degraded by poor radio reception, with preserved prosody but without decipherable words or phonemes. In each functional run, participants listened to six short passages from *Alice in Wonderland* in their native language (18 s each) and six passages of distorted speech (18 s each). Additionally, four fixation blocks (12 s each) were included in each run. The total duration of each run was 264 s. Each participant completed two runs of the task.

##### Articulation localizer.

Participants performed two tasks: an articulation task in which they were asked to repeat out loud a string of previously memorized syllables (“bła” [bwa], “sza” [ʂa], “tra” [tra]—all frequently occurring in Polish as word constituents, but meaningless in isolation), and a motor task in which they were asked to perform a simple motor sequence with the fingers of both hands (touching the thumb with their index, ring, and little finger, respectively). The localizer was based on the articulation > motor sequence contrast. In each functional run, participants completed six articulation and six finger tapping blocks (18 s each). Additionally, four fixation blocks (12 s each) were included in each run. Each participant completed two runs of the task.

##### Stroop localizer.

Participants were asked to overtly name the color of the presented word. The design of the task followed the one used by Fedorenko et al. (2011), in which participants completed blocks of the easy and hard versions of the task. In the easy condition, the words corresponded to non-color adjectives (“daleki” [“far”], “wysoki” [“tall”], “głęboki” [“deep”]); in the hard condition, the words corresponded to color adjectives (“niebieski” [“blue”], “zielony” [“green”], “czerwony” [“red”]). In the hard condition, half of the trials were conflict trials in which the word’s font color did not match its meaning; the other half were congruent trials. The order of trials within the hard condition was randomized. Each run started with a fixation cross (“+”) displayed on a gray background for 8,000 ms. Following that, participants completed 16 task blocks (8 per condition, 18 s each). Task blocks contained 12 trials which started with a fixation cross displayed for 500 ms followed by a word displayed for 1,000 ms. Additionally, five fixation blocks (18 s) were included in each run. The order of experimental and fixation blocks was counterbalanced between participants and runs (four counterbalance lists were created and each participant completed two of them). The localizer was based on the hard > easy condition contrast. The total duration of one run was 394 s. Each participant completed two runs of the task.

##### Multiple demand localizer.

Participants were asked to perform a spatial working memory task (e.g., Fedorenko et al., 2011; Fedorenko et al., 2013). The design of this task followed the one used by Fedorenko et al. (2011), in which the localizer contrast was based on the hard > easy condition contrast. In each trial, participants saw four 3 × 4 grids and were asked to memorize the locations of the fields that were marked as black on each of the grids. In the easy condition, they had to memorize one location per grid (four in total); in the hard condition, they had to keep track of two locations per grid (eight in total). After memorizing the grids, participants had to choose a grid that contained all the memorized locations from among two grids presented on the screen. Each trial started with a fixation cross displayed on the screen for 500 ms; this was followed by four grids, each displayed for 1,000 ms. After that, the choice task appeared on the screen until a response was given (for a max of 3,750 ms); this was followed by feedback (right or wrong) displayed for 250 ms and a fixation cross, which was displayed for 3,750 ms minus the reaction time in the choice task. In each of the conditions (easy and hard) participants completed four trials per block (34 s in total). Each run contained five easy condition blocks and five hard condition blocks. Additionally, six fixation blocks (16 s) were included in each run. The order of experimental and fixation blocks was counterbalanced between participants and runs (four counterbalance lists were created and each participant completed two of them). The total duration of one run was 438 s.

##### Verbal fluency localizer.

Within 10 s blocks, participants were asked to produce as many words as they could that either pertained to a given category (semantic fluency) or started with a given letter (phonemic fluency). The entire task was completed in L1. In each run, participants completed six semantic fluency and six phonemic fluency blocks, each focusing on a separate letter or semantic category. Each letter and category were presented only once, and they did not repeat between blocks and runs. A full list of stimuli used in this task is in the Supplementary Materials, available at https://doi.org/10.1162/nol_a_00128. In addition, each run included five baseline blocks, in which participants were asked to enumerate the months starting from January (Birn et al., 2010). The localizer contrast was based on task (semantic or phonemic fluency) > baseline condition contrast. Following Birn et al. (2010), each block was followed by a fixation cross displayed for 7–13 s. Each run lasted 348 s. Each participant completed two runs of the task.

### MRI Data Acquisition

MRI data were acquired using a 3T scanner (Magnetom Skyra, Siemens) with a 64-channel head coil. High-resolution, whole-brain anatomical images were acquired using a T1-weighted MPRAGE sequence (208 sagittal slices; voxel size 0.9 × 0.9 × 0.9 mm^3^; TR = 1,800 ms, TE = 2.32 ms, flip angle = 8°). A gradient field map (magnitude and phase images) was acquired with a dual-echo gradient-echo sequence, matched spatially with fMRI scans (TE1 = 4.92 ms, TE2 = 7.3 ms, TR = 503 ms, flip angle = 60°). Functional T2*-weighted images were acquired using a whole-brain echo-planar (EPI) pulse sequence (50 axial slices, 3 × 3 × 3 mm isotropic voxels; TR = 1,400 ms; TE = 27 ms; flip angle = 70°; field of view 192 mm; MB acceleration factor 2; in-plane GRAPPA acceleration factor 2 and phase encoding A >> P) using 2D multiband EPI sequences from the Center for Magnetic Resonance Research (CMRR), University of Minnesota (Xu et al., 2013). In order to account for magnetic saturation effects, the first four volumes of each sequence were not included in the analysis.

### MRI Data Preprocessing

All data were visually inspected for artifacts. The non-brain tissue was removed using the FSL Brain Extraction Tool (Smith, 2002). For the preprocessing of functional data, we used FSL FEAT (Version 6.0.0; Woolrich et al., 2001). The preprocessing steps included high-pass temporal filtering (100 s), spatial smoothing (full width at half maximum = 5 mm), distortion correction of functional images using fieldmap with FUGUE, co-registration and normalization using FLIRT (Jenkinson et al., 2002; Jenkinson & Smith, 2001) and motion correction using MCFLIRT (Jenkinson et al., 2002) with six rigid-body transformations. Functional images were co-registered with their anatomical images; subsequently, they were registered to MNI standard space (FSL’s 2 mm standard brain template).

### MRI Data Analysis

In the first-level statistical analysis, a double-gamma hemodynamic response function was used to model the blood oxygen level dependent (BOLD) signal corresponding to each event (trial in the main task, as it followed the event-related design; and blocks in the localizer tasks which followed a blocked design). The estimates of motion obtained with MCFLIRT (Jenkinson et al., 2002) were included in the first-level generalized linear model as nuisance covariates. For the localizer tasks, the two functional runs of each localizer task were combined in a second-level analysis for each participant using an FSL fixed-effect model. To ensure better comparability of our findings with previous studies, for language, MD, and articulation localizers we used sets of functional parcels established and validated by previous studies instead of creating group-level parcels based on our data (12 language parcels: Fedorenko et al., 2010; 20 MD parcels: Fedorenko et al., 2013; 11 articulation parcels: Basilakos et al., 2018). It is important to note that the group-level parcels from previous studies showed a high overlap with parcels for the language, MD, and articulation networks defined based on our data. For the remaining Stroop and verbal fluency localizers, group-constrained subject-specific regions of interest (ROIs) were defined for each participant following the approach proposed by Fedorenko et al. (2010). In the first step, the individual subject activation maps thresholded at 5% of most active voxels. In most cases this threshold was slightly more conservative than z > 3.1, uncorrected at the whole-brain level. We chose to use the 5% of most active voxels instead of a standard threshold because it yielded maps with comparable numbers of voxels between subjects that consequently produced more coherent group-level parcels. Subsequently, the individual parcels were binarized and overlaid on top of each other. The probabilistic map obtained this way was then thresholded so that only voxels active in more than five participants (∼20%) would be included. In the second step, the probabilistic maps were smoothed using an 8 × 8 × 8 Gaussian kernel and, subsequently, group-level partitions were created using a watershed algorithm for segmentation. This procedure yielded 19 group-level parcels for the Stroop task and 15 group-level parcels for the verbal fluency task. Before further analyses, we excluded very small parcels (mean size across subjects <10 voxels). This yielded a final set of 13 parcels within the Stroop network and 13 parcels within the verbal fluency network. In each of these parcels significant activation to the localizer contrast, that is, color words > neutral words for the Stroop task and fluency > automated speech for verbal fluency (thresholded at z > 2, corrected for multiple comparison using a cluster-wise correction at *p* < 0.05) was found in at least 51% of participants for the Stroop parcels (mean = 70.36%) and in at least 73.17% of participants for the verbal fluency parcels (mean = 84.99%). In the last step, group-level partitions were intersected with subject-specific data, thus yielding a set of subject-specific group-constrained ROIs. Finally, we selected the top 10% most responsive voxels within each parcel based on the *z* value map for a given localizer contrast (language: speech > degraded speech; MD: hard > easy visual working memory task; articulation: syllables > motor sequence; Stroop: color words > neutral words; and verbal fluency: fluency > automated speech), and we binarized the obtained ROIs. We then used the ROIs corresponding to all localizer tasks to extract parameter estimates corresponding to percent signal change in the two critical conditions of the main task: naming in L1 after L1, and naming in L1 after L2. Parameter estimates were extracted from the first-level analyses for each participant separately. For the analysis of the functional localizer scope, we used an across-run cross-validation procedure to extract the percent signal change corresponding to each localizer task condition (i.e., we defined the functional parcels based on the first functional run and estimated responses to the localizer contrast on the second run and repeated this procedure using the second run to define the functional parcels and the first run to extract response estimates; see Fedorenko et al., 2010). This was done using FSL’s FEATquery tool (https://www.FMRIb.ox.ac.uk/fsl/feat5/featquery.html). Further analyses were performed on the extracted parameter estimates in R (Version: 4.0.2; R Core Team, 2020). Following an approach that proposes modeling activity within a functional network on the network level instead of relying on separate analyses for each ROI (Malik-Moraleda et al., 2021), we fitted one linear mixed-effect model for each functional network according to the following formula:

$$\%\;\text{signal}\;\text{change}∼\text{PrecedingLanguage}+\left(1+\text{PrecedingLanguage}|\text{Subject}\right)+\left(1+\text{PrecedingLanguage}|\text{ROI}\right)$$

In each model, the fixed effect of PrecedingLanguage corresponded to a predictor with two levels: *L1 after L1* and *L1 after L2*. Before the analysis, this categorical predictor was deviation-coded (L1 after L1 = −0.5; L1 after L2 = 0.5). The analysis was performed using the lmer() function from the lmerTest package (Kuznetsova et al., 2017). For each functional network, we first fitted a maximal model and then identified the best random-effects structure following the recommendations of Bates et al. (2018). The reason for modeling the activity within each network with ROI as a random effect instead of a fixed effect relies on the assumption that the different brain regions engaged within a given functional network exhibit a significant degree of functional integration. To further explore the fine-grained differences between our critical conditions, which might only affect a subset of functional parcels within each network, we also fitted separate models for each ROI in each of the functional networks. The results of the ROI-specific analyses are reported in the Supplementary Materials. Raw neuroimaging data used in this study are freely available at the OpenNeuro repository (https://openneuro.org/datasets/ds004456). Data and code necessary to reproduce the ROI and behavioral analyses are freely available at https://osf.io/59za8/.

## RESULTS

### Functional Localizers’ Scope

**Figure 2.**
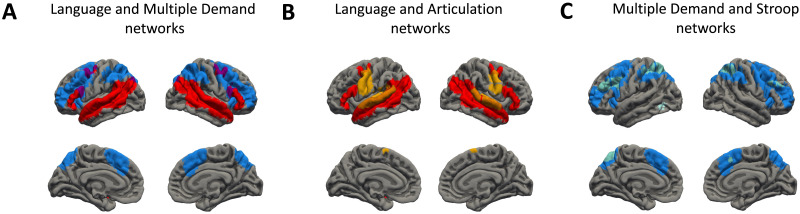
Brain masks corresponding to group-level functional networks. The figure presents a comparison of brain networks identified using different functional localizers: (A) language (red) and multiple demand (blue) networks; (B) language (red) and articulation (yellow) networks; (C) multiple demand (blue) and Stroop (turquoise) networks. All networks presented correspond to a combination of group-level parcels.

Functional networks identified with a set of localizer tasks were based on robust contrasts that yielded strong effects in each network (language localizer: intact vs. degraded speech: *β* = 0.520, *t*(40) = 5.684, *p* < 0.001; MD localizer: hard vs. easy visual working memory task: *β* = 0.580, *t*(40) = 13.12, *p* < 0.001; articulation localizer: articulation vs. motor task: *β* = 1.337, *t*(40) = 20.84, *p* < 0.001; Stroop localizer: Stroop task blocks vs. congruent color naming: *β* = 0.435, *t*(40) = 11.75; *p* < 0.001; verbal fluency localizer: fluency vs. enumerating months: *β* = 1.155, *t*(40) = 16.73, *p* < 0.001). The localizer tasks yielded five sets of parcels covering a wide range of brain regions. Language and MD parcels covered two robust nonoverlapping networks in the brain (Figure 2A; although small parts of language and MD network show some overlap, this was the case only for the group-level parcels, not for the individual subject-specific parcels). The articulation parcels included a set of regions partially overlapping with the language network in the temporal ROIs but also included parcels that selectively responded to articulation but not language (Figure 2B). As expected, the Stroop network largely overlapped with the MD network (for similar results see Fedorenko et al., 2013), however, the Stroop parcels were considerably smaller than the MD network (Figure 2C). The differences in size between the Stroop and the MD networks are partially driven by the fact that the Stroop parcels were created based on single-subject activation maps thresholded at 5% of the most active voxels (for details see Materials and Methods), which puts an a priori constraint on the size of the activation maps and the parcels that are created based on these maps. However, the Stroop network was also considerably smaller than the MD network when other, less stringent thresholds were used (e.g., z > 2). As such, the Stroop network can be considered a functional subsystem of the larger and more general MD network. Finally, the verbal fluency localizer yielded a set of strongly left-lateralized parcels partially overlapping both with the language and the MD networks (for verbal fluency parcels see Figure 4 in Digging Deeper: Specific Mechanisms of Bilingual Language Control, below).

To further characterize the functional networks as well as the scope of the localizer tasks used in this study, we ran additional analyses showing (1) responses to all localizer tasks used in this study within the language and the MD networks; and (2) responses to the language and MD localizer tasks (i.e., listening to degraded vs. intact speech and hard vs. easy working memory task) in all five functional networks used in this study. Results of these analyses can be found in the Supplementary Materials.

### Bilingual Language Control: Domain General or Language Specific

**Figure 3.**
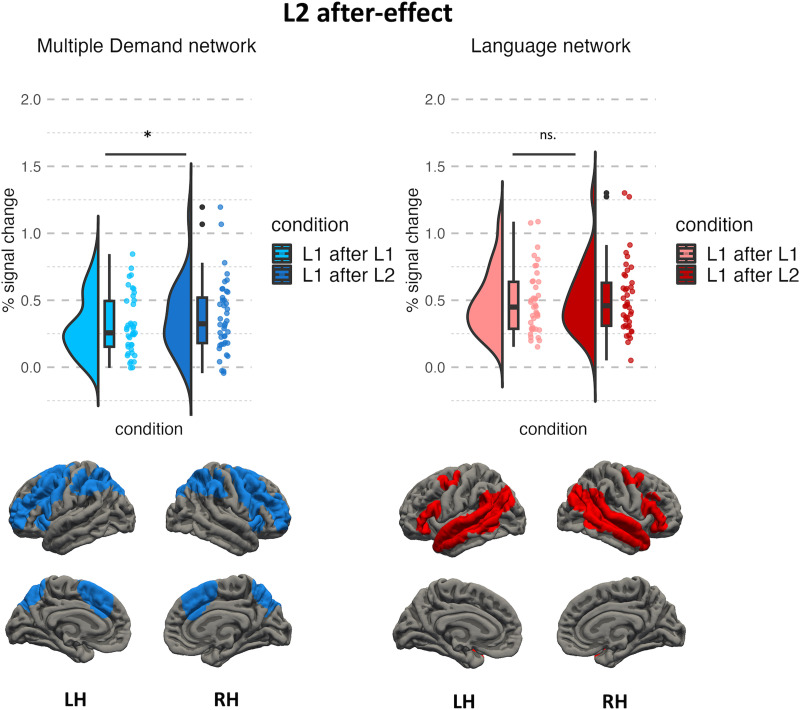
Neural activation corresponding to the L2 after-effect within the multiple demand and language networks. Plots represent % blood oxygen level dependent signal change in response to naming pictures in L1 after L1 (light hues) and L1 after L2 (dark hues). Rendered maps present sets of group-level parcels used in the subject-specific analysis. LH = left hemisphere, RH = right hemisphere.

To address the question of whether the bilingual language control mechanisms driving the L2 after-effect rely on domain-general or domain-specific mechanisms, we compared brain activity corresponding to naming pictures in L1 after L1 and L1 after L2 within two functional networks: the language network and the MD network. The results revealed no significant effect of the preceding language within the language network (*β* = 0.026, *t*(40) = 0.89, *p* = 0.379, effect size = 0.28); however, a significant effect of the preceding language was found within the MD network (*β* = 0.072, *t*(40) = 2.679, *p* = 0.011, effect size = 0.85). The results of the comparison between L1 after L1 and L1 after L2 within the language and MD networks are presented in Figure 3.

Separate analyses on each individual ROI showed significant differences between L1 after L1 and L1 after L2 in four ROIs in the MD network (left and right angular gyrus/superior parietal lobule, right superior and middle frontal gyrus, and right frontal pole), but no significant results were found in the language network. Full results showing comparisons between conditions for each ROI and each network are presented in Table S1 and Table S2 in the Supplementary Materials.

### Digging Deeper: Specific Mechanisms of Bilingual Language Control

**Figure 4.**
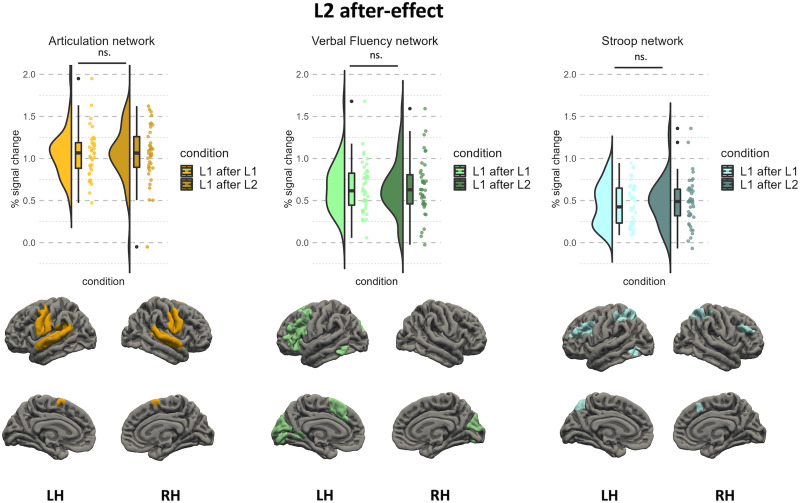
Neural activation corresponding to the L2 after-effect within the articulation, verbal fluency, and Stroop networks. Plots represent % blood oxygen level dependent signal change in response to naming pictures in L1 after L1 (bright hues) and L1 after L2 (dark hues). Rendered maps present sets of group-level parcels used in the subject-specific analysis. LH = left hemisphere, RH = right hemisphere.

To explore which specific mechanisms drive the L2 after-effect, we compared the brain activity corresponding to naming pictures in L1 after L1 and L1 after L2 within three functional networks identified with the articulation, Stroop, and verbal fluency tasks. We did not find a significant effect of the preceding language in any of the three networks (articulation: *β* = 0.005, *t*(40) = 0.09, *p* = 0.926, effect size = 0.03; Stroop: *β* = 0.034, *t*(40) = 1.02, *p* = 0.314, effect size = 0.32; verbal fluency: *β* = 0.003, *t*(40) = 0.07, *p* = 0.941, effect size = 0.02; see Figure 4). Separate analyses on each individual ROI for each of these networks are presented in Tables S3–S5 in the Supplementary Materials.

## DISCUSSION

### Bilingual Language Control: Domain General or Language Specific?

We explored the neurocognitive mechanisms underlying the L2 after-effect: a difficulty in producing words in the L1 after having used the L2. We compared brain activity corresponding to speaking in L1 after L2 with speaking in L1 after L1 within functionally defined brain networks. Our goal was to test (1) whether the L2 after-effect is related to activity in domain-general or domain-specific brain networks; and (2) whether the L2 after-effect reflects lexical retrieval difficulty, interference resolution, or increased difficulty at the phonological and articulatory stages of speech production. Unlike previous work exploring the neural basis of bilingual speech production, we used individual-level functional localizers (Fedorenko et al., 2010) to provide functional interpretations of brain responses associated with the L2 after-effect.

To address the question of whether the L2 after-effect is associated with increased engagement of language-specific and/or domain-general brain networks, we compared brain responses to speaking in L1 after L2 and L1 after L1 with two well-established, robust functional networks: the language network (Fedorenko et al., 2010; Malik-Moraleda et al., 2022; Scott et al., 2017) and the MD network (Duncan, 2010; Fedorenko et al., 2013). We found that the difficulty of naming pictures in L1 after L2 is related to increased BOLD responses within the domain-general MD network, but not within the domain-specific language network. Furthermore, to explore whether the L2 after-effect is driven by lexical access difficulty, increased interference between language-specific representations, or articulatory difficulty, we used three additional functional tasks as localizers: verbal fluency, the Stroop task, and an articulation task. We found that the L2 after-effect does not overlap with any of these process-specific localizers. We have also replicated the results of a behavioral study (Casado et al., 2022) showing that the magnitude of the L2 after-effect depends on the balance between languages, defined as a difference between mean naming latencies in L2 and L1 (see Supplementary Materials for a detailed report). This is an important note as our study is the first to record naming latencies corresponding to the L2 after-effect in the scanner. As such, our data provide straightforward evidence that the L2 after-effect can be successfully tested in the MRI scanner, despite the noisy and challenging environment.

### The L2 After-Effect Reflects the Increased Engagement of Domain General Cognitive Control

We found evidence for increased response for speech production in L1 after L2 compared to L1 after L1 in the MD network but not within the language network (the interaction between condition and network was, however, not significant (*β* = 0.046, *t*(2,470) = 1.89, *p* = 0.058). On the most general level, linking the L2 after-effect to increased activation in the MD system supports models that posit domain-general bilingual language control (Abutalebi & Green, 2016; Green & Abutalebi, 2013; Wu et al., 2019).

The MD network has been claimed to support cognitive control by integrating different types of information and binding them together to support the current cognitive operation (Assem et al., 2022; Assem, Glasser, et al., 2020; Cole et al., 2013; Koechlin & Summerfield, 2007). Activity within the MD network has been linked to mechanisms responsible for the implementation and reconfiguration of task sets (Dosenbach et al., 2006; Dosenbach et al., 2007), voluntary attentional control (goal-directed attention; Asplund et al., 2010; Corbetta & Shulman, 2002), and reorienting attention to novel tasks (Corbetta et al., 2008). An important feature of the MD network is that it is more engaged in less-automatized and untrained tasks (Shashidhara et al., 2019), thus implying that it implements a domain-general mechanism that supports any difficult or not sufficiently automatized activity that requires additional attentional support. Seen from this perspective, speaking in L1 after L2 may recruit additional domain-general resources to support the execution of a language task. However, this leaves open the question of why exactly speaking in L1 after L2 is so much more difficult than speaking in L1 after L1 that it requires additional support from the MD network.

### The L2 After-Effect Reflects Neither Word Retrieval Difficulties nor Increased Interference Between Language Specific Representations

The L2 after-effect has been previously explained within different theoretical frameworks (see Introduction). While these accounts provide plausible explanations of this effect, no univocal evidence in favor or against these interpretations has been provided (Casado et al., 2022; Declerck & Koch, 2023; Wodniecka, Szewczyk, et al., 2020). All available accounts of the L2 after-effect build on a common theoretical assumption that the difficulty in producing words in L1 after using L2 is a consequence of a control mechanism that changes the balance between the relative activation of L1 and L2 representations (Casado et al., 2022; for a discussion see Declerck & Koch, 2023). Importantly, according to these accounts the change in L1–L2 balance results in increased interference between the two languages that persists for some time after the speaker switches back to speaking in L1 (Casado et al., 2022), which, in turn, increases the difficulty of access and selection of lexicosemantic representations (e.g., Casado et al., 2022) and/or phonological representations and articulatory programs of L1 (e.g., Guo et al., 2011).

The results of the current study do not provide evidence supporting the notion that the L2 after-effect is related to increased interference at the lexicosemantic, phonological, or articulatory levels. We found that the L2 after-effect was not linked to an increased BOLD response within the core language network. Since lexical access and selection mechanisms pertain to core language computations (Hu et al., 2023), difficulties in lexical access should be reflected by an increased BOLD response within the language network. This was, however, not the case in the current study. What is more, we did not find increased brain response to speech production in the L1 after L2 condition in either of the functional networks that were identified using verbal fluency and articulation tasks. Even though both localizer tasks produced robust responses and allowed us to identify a number of functional ROIs, they did not overlap with the neural correlates of the L2 after-effect. Furthermore, we did not find any overlap between the neural correlates of the L2 after-effect and the functional network that was identified using the Stroop task; thus, we found no evidence for increased interference between language representations during speech production in L1 after L2.

The results of the localizer-based analyses provide a rather unexpected characterization of the neural correlates of the L2 after-effect. Contrary to the previous interpretations (Branzi et al., 2014; Branzi et al., 2016; Declerck & Koch, 2023; Guo et al., 2011; Misra et al., 2012), the current results indicate that the L2 after-effect reflects neither lexical access difficulty, nor articulatory difficulty, nor the additional engagement of language-specific mechanisms that support word retrieval. In other words, our results suggest that the L2 after-effect does not arise from within the language system itself (or from within language-specific representations). So, how can we then account for the increased difficulty in speaking in the native language after using L2? In the next section, we propose two putative explanations of the difficulty in production in L1 following the use of L2.

### Putative Explanations of the L2 After-Effect

#### The L2 after-effect as interference between task schemas

As described in the Introduction, most of the evidence for the involvement of cognitive control in bilingual processing comes from the language-switching paradigm and, more specifically, from (a) the asymmetrical language switch cost, that is, the finding that the cost of switching to the dominant (L1) language is bigger than to the weaker (L2) language; and (b) language mixing cost effects, that is, the stronger language (L1) suffers more when both languages are mixed within one block of naming (Costa & Santesteban, 2004; Gollan & Ferreira, 2009; Meuter & Allport, 1999; Verhoef et al., 2009; for review see Bobb & Wodniecka, 2013; Declerck et al., 2020; Declerck & Koch, 2023; Declerck & Philipp, 2015). Perhaps the most classic explanation of these effects assumes that they result from the proactive language control that is recruited whenever participants have to name a picture in the weaker L2. This control is recruited to “protect” the weaker L2 (less activated or less automatized) from interference with the stronger L1, either by inhibiting L1 (e.g., Green, 1998; Green & Abutalebi, 2013) or by bolstering the activation of L2 (e.g., Blanco-Elorrieta & Caramazza, 2021). When, after using L2, a bilingual speaker switches to their L1, naming in L1 is hampered by the consequences of prior control mechanisms: L1 is now either inhibited or there is an increased interference with L2, which is still strongly activated.

Explanations that assume the involvement of proactive control have been previously used to account for the L2 after-effect (Declerck & Koch, 2023; Wodniecka, Szewczyk, et al., 2020), and our current findings are generally consistent with this framework. However, they also point to an important caveat regarding the level of the targeted representations. While models of bilingual language processing typically assume that proactive control targets language-specific representations, such as specific lexical units (see, e.g., BIA+ model, Dijkstra & van Heuven, 2002, for bilingual language comprehension; Blanco-Elorrieta & Caramazza, 2021, for bilingual language production), the findings of the current study are incompatible with such views. If proactive control operated on language-specific representations, we should see modulations of brain activity in the after L1 vs. after L2 conditions in regions directly operating on these language-specific representations, namely in the language network, which was not the case.

Nevertheless, our results support accounts that assume proactive control if one assumes that the control does not target language-specific representations but rather more abstract ones. Previous literature claims the existence of such representations and refers to them as control representations (Braver et al., 2021) or hierarchical task schemas (Badre & Nee, 2018). Such representations could be conceived as sets of rules for orchestrating access to language-specific representations—the program we execute when we intend to speak in a given language and which guides all subprocesses involved in subsequent episodes of language production. This may perhaps be best understood in light of the cascade model of executive control (Koechlin & Summerfield, 2007), which proposes a hierarchy of increasingly abstract control processes. At the sensorimotor control level, a stimulus is simply associated with a reaction (e.g., picking up the phone when it rings). At the contextual control level, the stimulus–response link depends on the current context (not picking up the phone when it rings at your friend’s home). Episodic and branching control levels enable exceptions to the rules under which the current contextual rules can be temporarily and conditionally withheld or reinstated (not picking up the phone at a friend’s place unless he asked you to). Koechlin and Summerfield note that all dual-task situations require such high-level reconfigurations of rules (i.e., task 2 requires temporarily withholding the rule sets of task 1). Thus, when conceived from the perspective of this framework, the interference between L1 and L2 can occur at the level of rule sets that are managed by these more abstract levels of cognitive control. The interference occurs between the sets of task rules for speaking in L1 and the sets of task rules for speaking in L2, both of which compete for selection to guide language production. If the interference indeed occurs at the language task-schema level, it would explain why language representations, supported and processed by the language network, are not affected by a prior use of L2, yet it usually slows down the naming process (Branzi et al., 2014; Casado et al., 2022; Degani et al., 2020; Wodniecka, Szewczyk, et al., 2020). We therefore propose that the activation within the MD network may reflect the costs of resolving the interference between L1 and L2 production task schemas.

This idea is in line with the inhibitory control hypothesis (Green, 1998), which proposed that language-switching effects are a consequence of competition between language schemas. However, the inhibitory control hypothesis did not clearly define *language schemas*: they are sometimes described at a more abstract level as sets of rules that guide speech production in different languages, similar to what we propose here; at other times, they are framed as “regulating the outputs from the lexico-semantic system by altering the activation levels of representations within that system and by inhibiting outputs from that system” (Green, 1998), which implies that they engage in resolution of interference between language-specific representations.

Our proposal is also relevant to a distinction made in the literature regarding bilingual cognitive control, which differentiates between local and global language control. According to this distinction, bilingual language control mechanisms (e.g., inhibition) can target not only specific lexical representations (local control) but also the entire language system (global control: Branzi et al., 2014; Van Assche et al., 2013; Wodniecka, Szewczyk, et al., 2020). The idea that difficulty in bilingual speech production can be driven by increased interference between sets of rules that guide speech production in a given language aligns with the idea of global language control as mechanisms that operate not on language-specific representations (e.g., lemmas; cf. Van Assche et al., 2013; or nodes linked to a common language node; Blanco-Elorrieta & Caramazza, 2021; Dijkstra & van Heuven, 2002) but on sets of schemas for speaking in a given language.

In sum, while our first tentative explanation of the L2 after-effect builds on a mechanism that has been proposed before (proactive interference during L2 naming), it also points to a critical difference. The key novelty of the proposed interpretation is that the affected representations are not language-specific (lexical units, morphosyntactic units, phonological representations, motor programs) but more general and abstract task schemas that determine the general programs of using L1 or L2.

#### The L2 after-effect as sustained activation of cognitive control

Another possibility that, to the best of our knowledge, has not been proposed in the bilingual language processing literature, attributes the after-effects in blocked designs entirely to cognitive control mechanisms without involving any representations at the level of either language or task schema. This proposal is similar to the first explanation, because it also assumes that a prior speech production task in L2 requires intensified proactive cognitive control to resolve the interference between stronger L1 and weaker L2. However, unlike the first explanation, here we propose that the prior use of L2 leads to increased activation of cognitive control without ever impacting language-specific representations. Instead, the difficult task of speaking in L2 engages cognitive control so much that the control system remains alert even after the task in L2 concludes. We propose that this increased alertness might be achieved by increased engagement of the performance or error-monitoring systems in the brain. As such, the execution of any task (even a nonlinguistic one) would proceed in a more controlled way leading to an increase in reaction times.

Support for the explanation assuming that the L2 after-effect reflects persisting increased engagement of cognitive control may be found in the dual mechanisms of control framework (Braver et al., 2021). According to this framework, proactive control recruits the lateral prefrontal cortex, which, unlike the anterior cingulate cortex, is sensitive not only to errors or conflicts in the previous trial but also to control demands specific to the entire task, or the current communicative context (Blanco-Elorrieta & Caramazza, 2021; Casado et al., 2022). Since we did not observe modulations between the L1-after and L2-after conditions in the anterior cingulate cortex activity (see Supplementary Materials) but in the lateral prefrontal cortex, which is part of the MD system (by-ROI results in the MD network are available in Supplementary Materials), it can be argued that the L2 after-effect is a manifestation of proactive control.

In sum, the second tentative explanation of the increased brain response related to the L2 after-effect proposes that it reflects the increased engagement of cognitive control triggered by the previous use of L2 and that this engagement lingers during a subsequent task in L1. However, this hypothesis has never been directly tested and, as such, further experiments are needed to falsify it.

While the increased effort related to the L2 after-effect observed within the MD network can be putatively explained by increased engagement of proactive mechanisms engaged in performance or error-monitoring systems, other explanations of the observed effect cannot be ruled out. One alternative explanation of the increased engagement of the MD network is related to the specificity of the task used in the current study. We used a picture naming task, which requires producing single words. Previous studies on monolingual speakers have shown that the MD network responds more strongly to naming objects (i.e., producing isolated words) than to describing events presented with pictures (i.e., producing full sentences; Hu et al., 2023). Similar effects have also been found for language comprehension: The MD network was shown to respond more strongly to listening to lists of unconnected words than full sentences (Diachek et al., 2020). Altogether these results suggest that the MD network does not support the core aspects of language production. This does not rule out the possibility that the engagement of the domain-general network reflects increased engagement of nonlinguistic control processes in the production of words in L1 after L2 in contrast to L1 after L1; however, it may suggest that the L2 after-effect measured in a picture naming task may be “inflated” by the mere demands related to producing single, isolated words. As such, the effect might reflect a laboratory phenomenon that does not necessarily exemplify cognitive control mechanisms that bilinguals engage in more natural conversations of everyday language use. As such, further studies implementing more naturalistic paradigms that require bilinguals to produce more complex outputs are needed to separate the components driven by task-related demands from the actual engagement of cognitive control in the L2 after-effect.

## CONCLUSIONS

The novelty of our contribution is twofold: First, to identify the cognitive mechanisms that correspond to the neural signature of the L2 after-effect, we used the functional localizer approach (Fedorenko et al., 2010), which to the best of our knowledge has never been used in experiments looking at the brain basis of bilingual speech production. Second, unlike previous studies, our conclusions are based on a design that was optimized to measure the index of language control (i.e., the L2 after-effect) while reducing many possible confounds. We believe that the combination of precise analytical tools, carefully designed tasks, as well as the relatively large number of participants tested (*n* = 42) lead to new insights into the neurocognitive mechanisms of language control in bilinguals.

In contrast to the dominant theories and views in the field, we found that the L2 after-effect is unlikely to arise as a result of interference between language-specific representations in L1 and L2 because it does not overlap with the language network, or with any task-specific localizer that targets specific language processes (lexical access, articulation, language interference, and inhibition). Instead, we found that the L2 after-effect overlaps with the MD network and thus reflects a domain-general mechanism. We propose that the L2 after-effect may manifest as either increased interference between high-level task schemas used to orchestrate language use in L1 and L2, or as a lingering increase in the engagement of domain-general cognitive control mechanisms in speech production.

## ACKNOWLEDGMENTS

The authors would like to thank all members of our Psychology of Language and Bilingualism Laboratory LangUsta who contributed to the research project by collecting and coding the data, all participants who took part in the study, as well as Mike Timberlake, who proofread the text. We are very grateful to Evelina Fedorenko for inspiration and insightful comments on the design and results. This research was possible thanks to an OPUS grant from the National Science Center Poland.

## FUNDING INFORMATION

Zofia Wodniecka, Narodowe Centrum Nauki (https://dx.doi.org/10.13039/501100004281), Award ID: OPUS 2017/27/B/HS6/00959.

## AUTHOR CONTRIBUTIONS

**Agata Wolna**: Conceptualization: Equal; Data curation: Lead; Formal analysis: Lead; Investigation: Lead; Methodology: Equal; Software: Lead; Validation: Lead; Visualization: Lead; Writing – original draft: Lead; Writing – review & editing: Equal. **Jakub Szewczyk**: Conceptualization: Equal; Methodology: Equal; Supervision: Supporting; Visualization: Supporting; Writing – original draft: Equal; Writing – review & editing: Equal. **Michele Diaz**: Conceptualization: Supporting; Methodology: Supporting; Writing – review & editing: Equal. **Aleksandra Domagalik**: Investigation: Supporting; Methodology: Supporting; Resources: Equal; Software: Supporting; Writing – review & editing: Equal. **Marcin Szwed**: Conceptualization: Supporting; Methodology: Supporting; Writing – review & editing: Equal. **Zofia Wodniecka**: Conceptualization: Equal; Funding acquisition: Lead; Methodology: Equal; Project administration: Lead; Resources: Equal; Supervision: Lead; Visualization: Supporting; Writing – original draft: Equal; Writing – review & editing: Equal.

## DATA AND CODE AVAILABILITY STATEMENT

The raw neuroimaging data collected for this study along with the data on participants’ language experience are available at the OpenNeuro repository (https://openneuro.org/datasets/ds004456). Data and code necessary to reproduce the presented ROI and behavioral analyses are available at https://osf.io/59za8/.

## Supplementary Material



## TECHNICAL TERMS

Functional localizers: Experimental tasks used to identify brain networks supporting the execution of different cognitive tasks. They allow researchers to identify functional systems without referring to anatomical markers alone.
Bilingual language control: A postulated sub-type of cognitive control mechanisms specifically aimed to allow bilinguals to efficiently use and switch between the two languages they use.
Cognitive control: A set of cognitive mechanisms that support flexible adaptations of information processing and behavior to the current goals and task demands.
Multiple demand (MD) network: A bilateral frontoparietal network in the brain supporting cognitive control and executive functions.
Language network: A left-lateralized frontotemporal network in the brain supporting core aspects of language processing.

## References

[bib1] Abutalebi, J., & Green, D. (2007). Bilingual language production: The neurocognition of language representation and control. Journal of Neurolinguistics, 20(3), 242–275. 10.1016/j.jneuroling.2006.10.003

[bib2] Abutalebi, J., & Green, D. W. (2016). Neuroimaging of language control in bilinguals: Neural adaptation and reserve. Bilingualism: Language and Cognition, 19(4), 689–698. 10.1017/S1366728916000225

[bib3] Asplund, C. L., Todd, J. J., Snyder, A. P., & Marois, R. (2010). A central role for the lateral prefrontal cortex in goal-directed and stimulus-driven attention. Nature Neuroscience, 13(4), 507–512. 10.1038/nn.2509, 20208526 PMC2847024

[bib4] Assem, M., Blank, I. A., Mineroff, Z., Ademoğlu, A., & Fedorenko, E. (2020). Activity in the fronto-parietal multiple-demand network is robustly associated with individual differences in working memory and fluid intelligence. Cortex, 131, 1–16. 10.1016/j.cortex.2020.06.013, 32777623 PMC7530021

[bib5] Assem, M., Glasser, M. F., Van Essen, D. C., & Duncan, J. (2020). A domain-general cognitive core defined in multimodally parcellated human cortex. Cerebral Cortex, 30(8), 4361–4380. 10.1093/cercor/bhaa023, 32244253 PMC7325801

[bib6] Assem, M., Shashidhara, S., Glasser, M. F., & Duncan, J. (2022). Precise topology of adjacent domain-general and sensory-biased regions in the human brain. Cerebral Cortex, 32(12), 2521–2537. 10.1093/cercor/bhab362, 34628494 PMC9201597

[bib7] Badre, D., & Nee, D. E. (2018). Frontal cortex and the hierarchical control of behavior. Trends in Cognitive Sciences, 22(2), 170–188. 10.1016/j.tics.2017.11.005, 29229206 PMC5841250

[bib8] Basilakos, A., Smith, K. G., Fillmore, P., Fridriksson, J., & Fedorenko, E. (2018). Functional characterization of the human speech articulation network. Cerebral Cortex, 28(5), 1816–1830. 10.1093/cercor/bhx100, 28453613 PMC5907347

[bib9] Bates, D., Kliegl, R., Vasishth, S., & Baayen, H. (2018). Parsimonious mixed models. arXiv. 10.48550/arXiv.1506.04967

[bib10] Benn, Y., Ivanova, A. A., Clark, O., Mineroff, Z., Seikus, C., Silva, J. S., Varley, R., & Fedorenko, E. (2023). The language network is not engaged in object categorization. Cerebral Cortex, 33(19), 10380–10400. 10.1093/cercor/bhad289, 37557910 PMC10545444

[bib11] Birn, R. M., Kenworthy, L., Case, L., Caravella, R., Jones, T. B., Bandettini, P. A., & Martin, A. (2010). Neural systems supporting lexical search guided by letter and semantic category cues: A self-paced overt response fMRI study of verbal fluency. NeuroImage, 49(1), 1099–1107. 10.1016/j.neuroimage.2009.07.036, 19632335 PMC2832834

[bib12] Blanco-Elorrieta, E., & Caramazza, A. (2021). A common selection mechanism at each linguistic level in bilingual and monolingual language production. Cognition, 213, Article 104625. 10.1016/j.cognition.2021.104625, 33608129

[bib13] Blanco-Elorrieta, E., & Pylkkänen, L. (2016). Bilingual language control in perception versus action: MEG reveals comprehension control mechanisms in anterior cingulate cortex and domain-general control of production in dorsolateral prefrontal cortex. Journal of Neuroscience, 36(2), 290–301. 10.1523/JNEUROSCI.2597-15.2016, 26758823 PMC6602022

[bib14] Blanco-Elorrieta, E., & Pylkkänen, L. (2017). Bilingual language switching in the laboratory versus in the wild: The spatiotemporal dynamics of adaptive language control. Journal of Neuroscience, 37(37), 9022–9036. 10.1523/JNEUROSCI.0553-17.2017, 28821648 PMC5597983

[bib15] Blank, I., Kanwisher, N., & Fedorenko, E. (2014). A functional dissociation between language and multiple-demand systems revealed in patterns of BOLD signal fluctuations. Journal of Neurophysiology, 112(5), 1105–1118. 10.1152/jn.00884.2013, 24872535 PMC4122731

[bib16] Bobb, S. C., & Wodniecka, Z. (2013). Language switching in picture naming: What asymmetric switch costs (do not) tell us about inhibition in bilingual speech planning. Journal of Cognitive Psychology, 25(5), 568–585. 10.1080/20445911.2013.792822

[bib17] Branzi, F. M., Della Rosa, P. A., Canini, M., Costa, A., & Abutalebi, J. (2016). Language control in bilinguals: Monitoring and response selection. Cerebral Cortex, 26(6), 2367–2380. 10.1093/cercor/bhv052, 25838037

[bib18] Branzi, F. M., Martin, C. D., Abutalebi, J., & Costa, A. (2014). The after-effects of bilingual language production. Neuropsychologia, 52, 102–116. 10.1016/j.neuropsychologia.2013.09.022, 24144955

[bib19] Braver, T. S., Kizhner, A., Tang, R., Freund, M. C., & Etzel, J. A. (2021). The dual mechanisms of cognitive control project. Journal of Cognitive Neuroscience, 33(9), 1990–2015. 10.1162/jocn_a_01768, 34407191 PMC10069323

[bib20] Braver, T. S., Reynolds, J. R., & Donaldson, D. I. (2003). Neural mechanisms of transient and sustained cognitive control during task switching. Neuron, 39(4), 713–726. 10.1016/S0896-6273(03)00466-5, 12925284

[bib21] Broos, W. P. J., Duyck, W., & Hartsuiker, R. J. (2018). Are higher-level processes delayed in second language word production? Evidence from picture naming and phoneme monitoring. Language, Cognition and Neuroscience, 33(10), 1219–1234. 10.1080/23273798.2018.1457168

[bib22] Calabria, M., Costa, A., Green, D. W., & Abutalebi, J. (2018). Neural basis of bilingual language control. Annals of the New York Academy of Sciences, 1426(1), 221–235. 10.1111/nyas.13879, 29917244

[bib23] Casado, A., Szewczyk, J., Wolna, A., & Wodniecka, Z. (2022). The relative balance between languages predicts the degree of engagement of global language control. Cognition, 226, Article 105169. 10.1016/j.cognition.2022.105169, 35709626

[bib24] Chein, J. M., & Schneider, W. (2005). Neuroimaging studies of practice-related change: fMRI and meta-analytic evidence of a domain-general control network for learning. Cognitive Brain Research, 25(3), 607–623. 10.1016/j.cogbrainres.2005.08.013, 16242923

[bib25] Chen, X., Affourtit, J., Ryskin, R., Regev, T. I., Norman-Haignere, S., Jouravlev, O., Malik-Moraleda, S., Kean, H., Varley, R., & Fedorenko, E. (2023). The human language system, including its inferior frontal component in “Broca’s area,” does not support music perception. bioRxiv. 10.1101/2021.06.01.446439 PMC10505454 37005063

[bib26] Coderre, E. L., Smith, J. F., van Heuven, W. J. B., & Horwitz, B. (2016). The functional overlap of executive control and language processing in bilinguals. Bilingualism: Language and Cognition, 19(3), 471–488. 10.1017/S1366728915000188, 27695385 PMC5042330

[bib27] Cole, M. W., Reynolds, J. R., Power, J. D., Repovs, G., Anticevic, A., & Braver, T. S. (2013). Multi-task connectivity reveals flexible hubs for adaptive task control. Nature Neuroscience, 16(9), 1348–1355. 10.1038/nn.3470, 23892552 PMC3758404

[bib28] Cole, M. W., & Schneider, W. (2007). The cognitive control network: Integrated cortical regions with dissociable functions. NeuroImage, 37(1), 343–360. 10.1016/j.neuroimage.2007.03.071, 17553704

[bib29] Corbetta, M., Patel, G., & Shulman, G. L. (2008). The reorienting system of the human brain: From environment to theory of mind. Neuron, 58(3), 306–324. 10.1016/j.neuron.2008.04.017, 18466742 PMC2441869

[bib30] Corbetta, M., & Shulman, G. L. (2002). Control of goal-directed and stimulus-driven attention in the brain. Nature Reviews Neuroscience, 3(3), 201–215. 10.1038/nrn755, 11994752

[bib31] Costa, A. (2005). Lexical access in bilingual production. In J. F. Kroll & A. M. B. de Groot (Eds.), Handbook of bilingualism: Psycholinguistic approaches (pp. 308–325). Oxford University Press. 10.1093/oso/9780195151770.003.0018

[bib32] Costa, A., Miozzo, M., & Caramazza, A. (1999). Lexical selection in bilinguals: Do words in the bilingual’s two lexicons compete for selection? Journal of Memory and Language, 41(3), 365–397. 10.1006/jmla.1999.2651

[bib33] Costa, A., & Santesteban, M. (2004). Lexical access in bilingual speech production: Evidence from language switching in highly proficient bilinguals and L2 learners. Journal of Memory and Language, 50(4), 491–511. 10.1016/j.jml.2004.02.002

[bib34] Crittenden, B. M., & Duncan, J. (2014). Task difficulty manipulation reveals multiple demand activity but no frontal lobe hierarchy. Cerebral Cortex, 24(2), 532–540. 10.1093/cercor/bhs333, 23131804 PMC3888372

[bib35] Dale, A. M. (1999). Optimal experimental design for event-related fMRI. Human Brain Mapping, 8(2–3), 109–114. 10.1002/(SICI)1097-0193(1999)8:2/3<109::AID-HBM7>3.0.CO;2-W, 10524601 PMC6873302

[bib36] De Baene, W., Duyck, W., Brass, M., & Carreiras, M. (2015). Brain circuit for cognitive control is shared by task and language switching. Journal of Cognitive Neuroscience, 27(9), 1752–1765. 10.1162/jocn_a_00817, 25901448

[bib37] Declerck, M., Kleinman, D., & Gollan, T. H. (2020). Which bilinguals reverse language dominance and why? Cognition, 204, Article 104384. 10.1016/j.cognition.2020.104384, 32634738 PMC7494617

[bib38] Declerck, M., & Koch, I. (2023). The concept of inhibition in bilingual control. Psychological Review, 130(4), 953–976. 10.1037/rev0000367, 35420846

[bib39] Declerck, M., & Philipp, A. M. (2015). A review of control processes and their locus in language switching. Psychonomic Bulletin & Review, 22(6), 1630–1645. 10.3758/s13423-015-0836-1, 25917142

[bib40] Degani, T., Kreiner, H., Ataria, H., & Khateeb, F. (2020). The impact of brief exposure to the second language on native language production: Global or item specific? Applied Psycholinguistics, 41(1), 153–183. 10.1017/S0142716419000444

[bib41] DeLuca, V., Rothman, J., Bialystok, E., & Pliatsikas, C. (2019). Redefining bilingualism as a spectrum of experiences that differentially affects brain structure and function. Proceedings of the National Academy of Sciences, 116(15), 7565–7574. 10.1073/pnas.1811513116, 30914463 PMC6462104

[bib42] DeLuca, V., Rothman, J., Bialystok, E., & Pliatsikas, C. (2020). Duration and extent of bilingual experience modulate neurocognitive outcomes. NeuroImage, 204, Article 116222. 10.1016/j.neuroimage.2019.116222, 31557543

[bib43] Diachek, E., Blank, I., Siegelman, M., Affourtit, J., & Fedorenko, E. (2020). The domain-general multiple demand (MD) network does not support core aspects of language comprehension: A large-scale fMRI investigation. Journal of Neuroscience, 40(23), 4536–4550. 10.1523/JNEUROSCI.2036-19.2020, 32317387 PMC7275862

[bib44] Diamond, A. (2013). Executive functions. Annual Review of Psychology, 64, 135–168. 10.1146/annurev-psych-113011-143750, 23020641 PMC4084861

[bib45] Dijkstra, T., & van Heuven, W. J. B. (2002). The architecture of the bilingual word recognition system: From identification to decision. Bilingualism: Language and Cognition, 5(3), 175–197. 10.1017/S1366728902003012

[bib46] Dosenbach, N. U. F., Fair, D. A., Miezin, F. M., Cohen, A. L., Wenger, K. K., Dosenbach, R. A. T., Fox, M. D., Snyder, A. Z., Vincent, J. L., Raichle, M. E., Schlaggar, B. L., & Petersen, S. E. (2007). Distinct brain networks for adaptive and stable task control in humans. Proceedings of the National Academy of Sciences, 104(26), 11073–11078. 10.1073/pnas.0704320104, 17576922 PMC1904171

[bib47] Dosenbach, N. U. F., Visscher, K. M., Palmer, E. D., Miezin, F. M., Wenger, K. K., Kang, H. C., Burgund, E. D., Grimes, A. L., Schlaggar, B. L., & Petersen, S. E. (2006). A core system for the implementation of task sets. Neuron, 50(5), 799–812. 10.1016/j.neuron.2006.04.031, 16731517 PMC3621133

[bib48] Duncan, J. (2010). The multiple-demand (MD) system of the primate brain: Mental programs for intelligent behaviour. Trends in Cognitive Sciences, 14(4), 172–179. 10.1016/j.tics.2010.01.004, 20171926

[bib49] Fedeli, D., Del Maschio, N., Sulpizio, S., Rothman, J., & Abutalebi, J. (2021). The bilingual structural connectome: Dual-language experiential factors modulate distinct cerebral networks. Brain and Language, 220, Article 104978. 10.1016/j.bandl.2021.104978, 34171596

[bib50] Fedorenko, E. (2021). The early origins and the growing popularity of the individual-subject analytic approach in human neuroscience. Current Opinion in Behavioral Sciences, 40, 105–112. 10.1016/j.cobeha.2021.02.023

[bib51] Fedorenko, E., Behr, M. K., & Kanwisher, N. (2011). Functional specificity for high-level linguistic processing in the human brain. Proceedings of the National Academy of Sciences, 108(39), 16428–16433. 10.1073/pnas.1112937108, 21885736 PMC3182706

[bib52] Fedorenko, E., Duncan, J., & Kanwisher, N. (2013). Broad domain generality in focal regions of frontal and parietal cortex. Proceedings of the National Academy of Sciences, 110(41), 16616–16621. 10.1073/pnas.1315235110, 24062451 PMC3799302

[bib53] Fedorenko, E., Hsieh, P.-J., Nieto-Castañón, A., Whitfield-Gabrieli, S., & Kanwisher, N. (2010). New method for fMRI investigations of language: Defining ROIs functionally in individual subjects. Journal of Neurophysiology, 104(2), 1177–1194. 10.1152/jn.00032.2010, 20410363 PMC2934923

[bib54] Friesen, D. C., Edwards, K., & Lamoureux, C. (2021). Predictors of verbal fluency performance in monolingual and bilingual children: The interactive role of English receptive vocabulary and fluid intelligence. Journal of Communication Disorders, 89, Article 106074. 10.1016/j.jcomdis.2020.106074, 33450631

[bib55] Frost, M. A., & Goebel, R. (2012). Measuring structural–functional correspondence: Spatial variability of specialised brain regions after macro-anatomical alignment. NeuroImage, 59(2), 1369–1381. 10.1016/j.neuroimage.2011.08.035, 21875671

[bib56] Gollan, T. H., & Ferreira, V. S. (2009). Should I stay or should I switch? A cost–benefit analysis of voluntary language switching in young and aging bilinguals. Journal of Experimental Psychology: Learning, Memory, and Cognition, 35(3), 640–665. 10.1037/a0014981, 19379041 PMC2864120

[bib57] Gollan, T. H., Kleinman, D., & Wierenga, C. E. (2014). What’s easier: Doing what you want, or being told what to do? Cued versus voluntary language and task switching. Journal of Experimental Psychology: General, 143(6), 2167–2195. 10.1037/a0038006, 25313951 PMC4244272

[bib58] Green, D. W. (1998). Mental control of the bilingual lexico-semantic system. Bilingualism: Language and Cognition, 1(2), 67–81. 10.1017/S1366728998000133

[bib59] Green, D. W., & Abutalebi, J. (2013). Language control in bilinguals: The adaptive control hypothesis. Journal of Cognitive Psychology, 25(5), 515–530. 10.1080/20445911.2013.796377, 25077013 PMC4095950

[bib60] Guo, T., Liu, H., Misra, M., & Kroll, J. F. (2011). Local and global inhibition in bilingual word production: fMRI evidence from Chinese–English bilinguals. NeuroImage, 56(4), 2300–2309. 10.1016/j.neuroimage.2011.03.049, 21440072 PMC3741343

[bib61] Haman, E., Łuniewska, M., & Pomiechowska, B. (2015). Designing cross-linguistic lexical tasks (CLTs) for bilingual preschool children. In S. Armon-Lotem, J. de Jong, & N. Meir (Eds.), Assessing multilingual children (pp. 196–240). Multilingual Matters. 10.21832/9781783093137-010

[bib62] Hu, J., Small, H., Kean, H., Takahashi, A., Zekelman, L., Kleinman, D., Ryan, E., Nieto-Castañón, A., Ferreira, V., & Fedorenko, E. (2023). Precision fMRI reveals that the language-selective network supports both phrase-structure building and lexical access during language production. Cerebral Cortex, 33(8), 4384–4404. 10.1093/cercor/bhac350, 36130104 PMC10110436

[bib63] Ivanova, A. A., Mineroff, Z., Zimmerer, V., Kanwisher, N., Varley, R., & Fedorenko, E. (2021). The language network is recruited but not required for nonverbal event semantics. Neurobiology of Language, 2(2), 176–201. 10.1162/nol_a_00030, 37216147 PMC10158592

[bib64] Jenkinson, M., Bannister, P., Brady, M., & Smith, S. (2002). Improved optimization for the robust and accurate linear registration and motion correction of brain images. NeuroImage, 17(2), 825–841. 10.1006/nimg.2002.1132, 12377157

[bib65] Jenkinson, M., & Smith, S. (2001). A global optimisation method for robust affine registration of brain images. Medical Image Analysis, 5(2), 143–156. 10.1016/S1361-8415(01)00036-6, 11516708

[bib66] Koch, I., Gade, M., Schuch, S., & Philipp, A. M. (2010). The role of inhibition in task switching: A review. Psychonomic Bulletin & Review, 17(1), 1–14. 10.3758/PBR.17.1.1, 20081154

[bib67] Koechlin, E., & Summerfield, C. (2007). An information theoretical approach to prefrontal executive function. Trends in Cognitive Sciences, 11(6), 229–235. 10.1016/j.tics.2007.04.005, 17475536

[bib68] Kroll, J. F., Bobb, S. C., & Wodniecka, Z. (2006). Language selectivity is the exception, not the rule: Arguments against a fixed locus of language selection in bilingual speech. Bilingualism: Language and Cognition, 9(2), 119–135. 10.1017/S1366728906002483

[bib69] Kuznetsova, A., Brockhoff, P. B., & Christensen, R. H. B. (2017). lmerTest package: Tests in linear mixed effects models. Journal of Statistical Software, 82(13), 1–26. 10.18637/jss.v082.i13

[bib70] La Heij, W. (2005). Selection processes in monolingual and bilingual lexical access. In J. F. Kroll & A. M. B. de Groot (Eds.), Handbook of bilingualism: Psycholinguistic approaches (pp. 289–307). Oxford University Press. 10.1093/oso/9780195151770.003.0017

[bib71] Lemhöfer, K., & Broersma, M. (2012). Introducing LexTALE: A quick and valid Lexical Test for Advanced Learners of English. Behavior Research Methods, 44(2), 325–343. 10.3758/s13428-011-0146-0, 21898159 PMC3356522

[bib72] Liljeström, M., Kujala, J., Stevenson, C., & Salmelin, R. (2015). Dynamic reconfiguration of the language network preceding onset of speech in picture naming. Human Brain Mapping, 36(3), 1202–1216. 10.1002/hbm.22697, 25413681 PMC4365727

[bib73] Luk, G., Green, D. W., Abutalebi, J., & Grady, C. (2012). Cognitive control for language switching in bilinguals: A quantitative meta-analysis of functional neuroimaging studies. Language and Cognitive Processes, 27(10), 1479–1488. 10.1080/01690965.2011.613209, 24795491 PMC4006828

[bib74] Malik-Moraleda, S., Ayyash, D., Gallée, J., Affourtit, J., Hoffmann, M., Mineroff, Z., Jouravlev, O., & Fedorenko, E. (2022). An investigation across 45 languages and 12 language families reveals a universal language network. Nature Neuroscience, 25(8), 1014–1019. 10.1038/s41593-022-01114-5, 35856094 PMC10414179

[bib75] Malik-Moraleda, S., Cucu, T., Lipkin, B., & Fedorenko, E. (2021). The domain-general multiple demand network is more active in early balanced bilinguals than monolinguals during executive processing. Neurobiology of Language, 2(4), 647–664. 10.1162/nol_a_00058, 37214622 PMC10158558

[bib76] Martin, A., Wiggs, C. L., Lalonde, F., & Mack, C. (1994). Word retrieval to letter and semantic cues: A double dissociation in normal subjects using interference tasks. Neuropsychologia, 32(12), 1487–1494. 10.1016/0028-3932(94)90120-1, 7885578

[bib77] Mendoza, M. N., Blumenfeld, H. K., Knight, R. T., & Ries, S. K. (2021). Investigating the link between linguistic and non-linguistic cognitive control in bilinguals using Laplacian-transformed event related potentials. Neurobiology of Language, 2(4), 605–627. 10.1162/nol_a_00056, 35243348 PMC8886518

[bib78] Meuter, R. F. I., & Allport, A. (1999). Bilingual language switching in naming: Asymmetrical costs of language selection. Journal of Memory and Language, 40(1), 25–40. 10.1006/jmla.1998.2602

[bib79] Michon, K. J., Khammash, D., Simmonite, M., Hamlin, A. M., & Polk, T. A. (2022). Person-specific and precision neuroimaging: Current methods and future directions. NeuroImage, 263, Article 119589. 10.1016/j.neuroimage.2022.119589, 36030062

[bib80] Miller, E. K., & Cohen, J. D. (2001). An integrative theory of prefrontal cortex function. Annual Review of Neuroscience, 24, 167–202. 10.1146/annurev.neuro.24.1.167, 11283309

[bib81] Misra, M., Guo, T., Bobb, S. C., & Kroll, J. F. (2012). When bilinguals choose a single word to speak: Electrophysiological evidence for inhibition of the native language. Journal of Memory and Language, 67(1), 224–237. 10.1016/j.jml.2012.05.001, 24222718 PMC3820915

[bib82] Nee, D. E., Jonides, J., & Berman, M. G. (2007). Neural mechanisms of proactive interference-resolution. NeuroImage, 38(4), 740–751. 10.1016/j.neuroimage.2007.07.066, 17904389 PMC2206737

[bib83] Niendam, T. A., Laird, A. R., Ray, K. L., Dean, Y. M., Glahn, D. C., & Carter, C. S. (2012). Meta-analytic evidence for a superordinate cognitive control network subserving diverse executive functions. Cognitive, Affective, & Behavioral Neuroscience, 12(2), 241–268. 10.3758/s13415-011-0083-5, 22282036 PMC3660731

[bib84] Pallier, C., Dehaene, S., Poline, J.-B., LeBihan, D., Argenti, A.-M., Dupoux, E., & Mehler, J. (2003). Brain imaging of language plasticity in adopted adults: Can a second language replace the first? Cerebral Cortex, 13(2), 155–161. 10.1093/cercor/13.2.155, 12507946

[bib85] Poldrack, R. A. (2006). Can cognitive processes be inferred from neuroimaging data? Trends in Cognitive Sciences, 10(2), 59–63. 10.1016/j.tics.2005.12.004, 16406760

[bib86] R Core Team. (2020). R: A language and environment for statistical computing (Version 4.0.2) [Software]. R Foundation for Statistical Computing. https://www.R-project.org/

[bib87] Reverberi, C., Kuhlen, A. K., Seyed-Allaei, S., Greulich, R. S., Costa, A., Abutalebi, J., & Haynes, J.-D. (2018). The neural basis of free language choice in bilingual speakers: Disentangling language choice and language execution. NeuroImage, 177, 108–116. 10.1016/j.neuroimage.2018.05.025, 29753107

[bib88] Rossi, E., Newman, S., Kroll, J. F., & Diaz, M. T. (2018). Neural signatures of inhibitory control in bilingual spoken production. Cortex, 108, 50–66. 10.1016/j.cortex.2018.07.009, 30130633 PMC6375513

[bib89] Runnqvist, E., Strijkers, K., Alario, F.-X., & Costa, A. (2012). Cumulative semantic interference is blind to language: Implications for models of bilingual speech production. Journal of Memory and Language, 66(4), 850–869. 10.1016/j.jml.2012.02.007

[bib90] Scott, T. L., Gallée, J., & Fedorenko, E. (2017). A new fun and robust version of an fMRI localizer for the frontotemporal language system. Cognitive Neuroscience, 8(3), 167–176. 10.1080/17588928.2016.1201466, 27386919

[bib91] Shain, C., Paunov, A., Chen, X., Lipkin, B., & Fedorenko, E. (2023). No evidence of theory of mind reasoning in the human language network. Cerebral Cortex, 33(10), 6299–6319. 10.1093/cercor/bhac505, 36585774 PMC10183748

[bib92] Shao, Z., Janse, E., Visser, K., & Meyer, A. S. (2014). What do verbal fluency tasks measure? Predictors of verbal fluency performance in older adults. Frontiers in Psychology, 5, Article 772. 10.3389/fpsyg.2014.00772, 25101034 PMC4106453

[bib93] Shashidhara, S., Mitchell, D. J., Erez, Y., & Duncan, J. (2019). Progressive recruitment of the frontoparietal multiple-demand system with increased task complexity, time pressure, and reward. Journal of Cognitive Neuroscience, 31(11), 1617–1630. 10.1162/jocn_a_01440, 31274390 PMC7116493

[bib94] Smith, D. M., Perez, D. C., Porter, A., Dworetsky, A., & Gratton, C. (2021). Light through the fog: Using precision fMRI data to disentangle the neural substrates of cognitive control. Current Opinion in Behavioral Sciences, 40, 19–26. 10.1016/j.cobeha.2020.12.004, 33553511 PMC7861476

[bib95] Smith, S. M. (2002). Fast robust automated brain extraction. Human Brain Mapping, 17(3), 143–155. 10.1002/hbm.10062, 12391568 PMC6871816

[bib96] Stroop, J. R. (1935). Studies of interference in serial verbal reactions. Journal of Experimental Psychology, 18(6), 643–662. 10.1037/h0054651

[bib97] Sulpizio, S., Del Maschio, N., Fedeli, D., & Abutalebi, J. (2020). Bilingual language processing: A meta-analysis of functional neuroimaging studies. Neuroscience & Biobehavioral Reviews, 108, 834–853. 10.1016/j.neubiorev.2019.12.014, 31838193

[bib98] Tahmasebi, A. M., Davis, M. H., Wild, C. J., Rodd, J. M., Hakyemez, H., Abolmaesumi, P., & Johnsrude, I. S. (2012). Is the link between anatomical structure and function equally strong at all cognitive levels of processing? Cerebral Cortex, 22(7), 1593–1603. 10.1093/cercor/bhr205, 21893681

[bib99] Unsworth, N., Spillers, G. J., & Brewer, G. A. (2011). Variation in verbal fluency: A latent variable analysis of clustering, switching, and overall performance. Quarterly Journal of Experimental Psychology, 64(3), 447–466. 10.1080/17470218.2010.505292, 20839136

[bib100] Van Assche, E., Duyck, W., & Gollan, T. H. (2013). Whole-language and item-specific control in bilingual language production. Journal of Experimental Psychology: Learning, Memory, and Cognition, 39(6), 1781–1792. 10.1037/a0032859, 23647380

[bib101] Verhoef, K., Roelofs, A., & Chwilla, D. J. (2009). Role of inhibition in language switching: Evidence from event-related brain potentials in overt picture naming. Cognition, 110(1), 84–99. 10.1016/j.cognition.2008.10.013, 19084830

[bib102] Weissberger, G. H., Gollan, T. H., Bondi, M. W., Clark, L. R., & Wierenga, C. E. (2015). Language and task switching in the bilingual brain: Bilinguals are staying, not switching, experts. Neuropsychologia, 66, 193–203. 10.1016/j.neuropsychologia.2014.10.037, 25446970 PMC4596720

[bib103] Wodniecka, Z., Casado, A., Kałamała, P., Marecka, M., Timmer, K., & Wolna, A. (2020). The dynamics of language experience and how it affects language and cognition. In K. D. Federmeier & H.-W. Huang (Eds.), Psychology of learning and motivation (Vol. 72, pp. 235–281). Academic Press. 10.1016/bs.plm.2020.02.005

[bib104] Wodniecka, Z., Szewczyk, J., Kałamała, P., Mandera, P., & Durlik, J. (2020). When a second language hits a native language. What ERPs (do and do not) tell us about language retrieval difficulty in bilingual language production. Neuropsychologia, 141, Article 107390. 10.1016/j.neuropsychologia.2020.107390, 32057934

[bib105] Wolna, A., Łuniewska, M., Haman, E., & Wodniecka, Z. (2023). Polish norms for a set of colored drawings of 168 objects and 146 actions with predictors of naming performance. Behavior Research Methods, 55(5), 2706–2732. 10.3758/s13428-022-01923-3, 35915359 PMC10439080

[bib106] Woolrich, M. W., Ripley, B. D., Brady, M., & Smith, S. M. (2001). Temporal autocorrelation in univariate linear modeling of fMRI data. NeuroImage, 14(6), 1370–1386. 10.1006/nimg.2001.0931, 11707093

[bib107] Wu, J., Yang, J., Chen, M., Li, S., Zhang, Z., Kang, C., Ding, G., & Guo, T. (2019). Brain network reconfiguration for language and domain-general cognitive control in bilinguals. NeuroImage, 199, 454–465. 10.1016/j.neuroimage.2019.06.022, 31200066

[bib108] Xu, J., Moeller, S., Auerbach, E. J., Strupp, J., Smith, S. M., Feinberg, D. A., Yacoub, E., & Uğurbil, K. (2013). Evaluation of slice accelerations using multiband echo planar imaging at 3T. NeuroImage, 83, 991–1001. 10.1016/j.neuroimage.2013.07.055, 23899722 PMC3815955

[bib109] Zhang, Y., Wang, T., Huang, P., Li, D., Qiu, J., Shen, T., & Xie, P. (2015). Free language selection in the bilingual brain: An event-related fMRI study. Scientific Reports, 5, Article 11704. 10.1038/srep11704, 26177885 PMC4503947

